# Supplementary material for: Recruitment of Corticotropin-Releasing Hormone (CRH) Neurons in Categorically Distinct Stress Reactions in the Mouse Brain
Source: Int J Mol Sci. 2023 Jul 21;24(14):11736. doi: 10.3390/ijms241411736 (PMC10380650; doi:10.3390/ijms241411736)
Supplement: Supplementary file 1 [file ijms-24-11736-s001.zip › ijms-2513829-supplementary.pdf]

**Supplementary figures:**

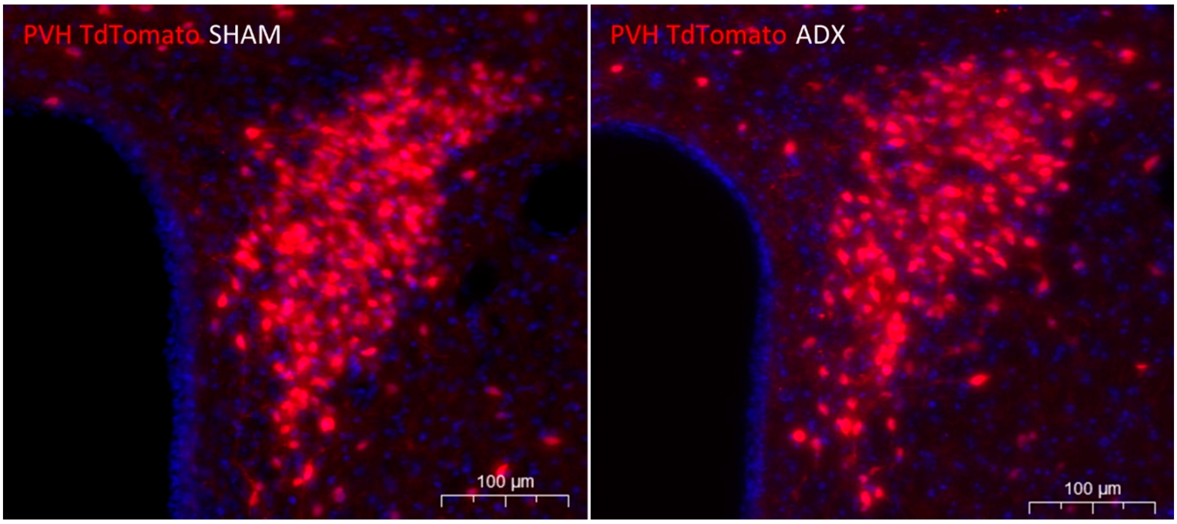


**Suppl. Figure S1.** TdTomato staining in the hypothalamic paraventricular nucleus (PVH) of sham operated (SHAM) and adrenalectomized (ADX) Crh-IRES-Cre;Ai9 mice.

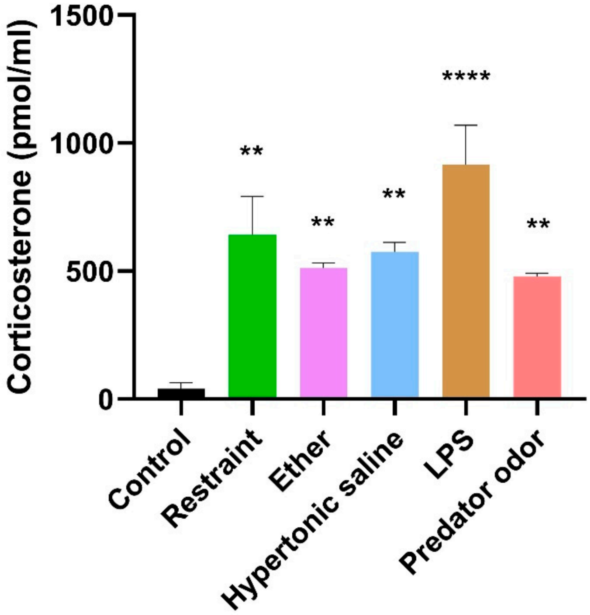


**Suppl. Figure S2.** Mean ± SEM values of plasma corticosterone in animals exposed to different stressors. **p<0.01; ****p<0.0001 compared to unstressed control.

(A)

| | NE | IP saline |
|---|---|---|
| MOBgl | ns | ns |
| MOBopl | ns | ns |
| AON | ns | ns |
| TTv | ns | ns |
| DP | ns | ns |
| DTr | ns | ns |
| PIR | ns | ns |
| COA | ns | ns |
| NLOT | ns | ns |
| O | ns | ns |
| MOs | ns | ns |
| AId | ns | ns |
| Mop | ns | * |
| LVOV | ns | ns |
| SSp | ns | ns |
| PL | ns | ns |
| ILA | * | ns |
| GU | * | ns |
| SSs | ns | ns |
| ACA | ns | ns |
| RSP | ns | ns |
| CA | ns | ns |
| DG | ns | ns |
| CA3 | ns | ns |
| Epd | ns | ns |
| CLA | ns | ns |
| LS | ns | ns |
| FS | ns | ns |
| OT | ns | ns |
| CEA | ns | ns |
| MEA | ns | ns |
| BSTa | ns | ns |
| BSTad | ns | ns |
| BSTav | ns | ns |
| BSTp | ns | ns |
| BAC | ns | ns |
| PMTH | ns | ns |
| PIL | ns | ns |
| G | ns | ns |
| RH | * | ns |
| CM | * | ns |
| RE | ns | ns |
| PVTa | ns | ns |
| PVTp | ns | ns |
| CL | * | ns |
| LGv | ns | ns |
| PeF | ns | ns |
| MCLHD | ns | ns |
| MCLHV | ns | ns |
| PVR | ns | ns |
| MPN | ns | ns |
| SCH | ns | ns |
| SO | ns | ns |
| PVH | ** | ns |
| AHNc | ns | ns |
| AHNp | *** | ns |
| LHA | ns | ns |
| VMH | ns | ns |
| ARH | ns | ns |
| STN | ns | ns |
| DMH | *** | ns |
| PHd | ns | ns |
| PHv | ** | ns |
| PSTN | ns | ns |
| PM | ns | ns |
| PHp | ** | ns |
| SUM | ns | ns |
| ZI | ns | ns |
| DpMe | ns | ns |
| PRC | * | ns |
| PAG | ns | ns |
| EW | ns | ns |
| SCs | ns | ns |
| IF | ns | ns |
| IPN | ns | ns |
| PG | ns | ns |
| PGdm | ns | * |
| TRN | ns | ns |
| CS | ns | ns |
| PCG | ns | ns |
| LC | ns | ns |
| B | ns | ns |
| PRP | ns | ns |
| MV | ns | ns |
| CN | ns | ns |
| X | ns | ns |
| IO | ns | ns |
| RPA | ** | ns |
| DMX | ns | ns |
| AP | ns | ns |
| NTS | ns | ns |
| ECU | ns | ns |
| CU | ns | ns |
| LRN | ns | ns |
| XII | ns | * |

(B)

| | NE | IP saline |
|---|---|---|
| MOBgl | ns | ns |
| MOBopl | ns | ns |
| AON | ns | ns |
| TTv | ns | ns |
| DP | ns | ns |
| DTr | ns | ns |
| PIR | ns | ns |
| COA | ns | ns |
| NLOT | ns | ns |
| O | ns | ns |
| MOs | ns | ns |
| AId | ns | ns |
| Mop | ns | ns |
| LVOV | ns | ns |
| SSp | ns | ns |
| PL | ns | ns |
| ILA | ns | ns |
| GU | ns | ns |
| SSs | ns | ns |
| ACA | ns | ns |
| RSP | ns | ns |
| CA | ns | ns |
| DG | ns | ns |
| CA3 | ns | ns |
| Epd | ns | ns |
| CLA | ns | ns |
| LS | ns | ns |
| FS | ns | ns |
| OT | ns | ns |
| CEA | ns | ns |
| MEA | ns | ns |
| BSTa | ns | ns |
| BSTad | ns | ns |
| BSTav | ns | ns |
| BSTp | ns | ns |
| BAC | ns | ns |
| PMTH | ns | ns |
| PIL | ns | ns |
| G | ns | ns |
| RH | ns | ns |
| CM | *** | ns |
| RE | ns | ns |
| PVTa | ns | ns |
| PVTp | ns | ns |
| CL | * | ns |
| LGv | ns | ns |
| PeF | * | ns |
| MCLHD | ns | * |
| MCLHV | ns | ns |
| PVR | ns | ns |
| MPN | ns | ns |
| SCH | ns | ns |
| SO | ns | ns |
| PVH | *** | ns |
| AHNc | ns | ns |
| AHNp | ns | ns |
| LHA | ns | ns |
| VMH | ns | * |
| ARH | ns | ns |
| STN | ns | ns |
| DMH | ns | ns |
| PHd | ns | ns |
| PHv | ns | ns |
| PSTN | ns | ns |
| PM | ns | ns |
| PHp | ns | ns |
| SUM | ns | ns |
| ZI | ns | ns |
| DpMe | ns | ns |
| PRC | ns | ns |
| PAG | ns | ns |
| EW | ns | ns |
| SCs | ns | ns |
| IF | ns | ns |
| IPN | ns | ns |
| PG | ns | ns |
| PGdm | ns | ns |
| TRN | ns | ns |
| CS | ns | ns |
| PCG | ns | ns |
| LC | ns | ns |
| B | ns | ns |
| PRP | * | ns |
| MV | ns | ns |
| CN | ns | ns |
| X | ns | ns |
| IO | ns | ns |
| RPA | ns | ns |
| DMX | ns | ns |
| AP | ns | ns |
| NTS | ns | ns |
| ECU | ns | ns |
| CU | * | ns |
| LRN | ns | ns |
| XII | ns | ns |

| |
|---|
| Olfactory areas |
| Isocortex |
| Hippocampal formation |
| CTXsp |
| Striatum |
| Pallidum |
| Thalamus |
| Hypothalamus |
| Midbrain |
| Pons |
| Medulla |

**Suppl. Figure S3.** Significance levels of differences seen between animals exposed to novel environment (NE) or intraperitoneal injection of saline and non-stressed (absolute) controls. (A) neuronal activation (FOS) and (B) colocalization (FOS + tdTomato). p*<0.05; p**<0.01; p***<0.001; ns= not significant.
